# Lignin from Micro- to Nanosize: Applications

**DOI:** 10.3390/ijms18112367

**Published:** 2017-11-08

**Authors:** Stefan Beisl, Anton Friedl, Angela Miltner

**Affiliations:** Institute of Chemical, Environmental and Biological Engineering, TU Wien, 1060 Vienna, Austria; anton.friedl@tuwien.ac.at (A.F.); angela.miltner@tuwien.ac.at (A.M.)

**Keywords:** lignin, nanoparticles, microparticles, biorefinery, reinforcing, antibacterial, UV-blocker, drug carrier, nanocomposites

## Abstract

Micro- and nanosize lignin has recently gained interest due to improved properties compared to standard lignin available today. As the second most abundant biopolymer after cellulose, lignin is readily available but used for rather low-value applications. This review focuses on the application of micro- and nanostructured lignin in final products or processes that all show potential for high added value. The fields of application are ranging from improvement of mechanical properties of polymer nanocomposites, bactericidal and antioxidant properties and impregnations to hollow lignin drug carriers for hydrophobic and hydrophilic substances. Also, a carbonization of lignin nanostructures can lead to high-value applications such as use in supercapacitors for energy storage. The properties of the final product depend on the surface properties of the nanomaterial and, therefore, on factors like the lignin source, extraction method, and production/precipitation methods, as discussed in this review.

## 1. Introduction

Lignocellulosic biomass residues are estimated to exceed 2 × 10^11^ t/year worldwide and offer a vast source for lignin [1]. Most of the lignin is currently used as an energy source. However, economic analysis has proved that the use of biomass for energy applications alone, in many cases, is not economically viable and utilization of the entire biomass through multiple processes is needed to improve the economics [2]. Only about 40% of the generated lignin is needed to cover the internal energy demand of a biorefinery [3,4]. Hence, 60% of the generated lignin is available to maximize valorization in addition to the valorization of the carbohydrate fractions.

Lignin in nanoscale can be one of the keys to maximize valorization of the input feedstock in a biorefinery, and “you can make anything you want out of lignin… except money” [5] is changing to “Yes, we can make money out of lignin” [6]. For example, lignin-derived carbon nanofibers were successfully produced in pilot scale at Scion and Revolution Fibres (Auckland, New Zealand) and offer low production cost relative to other carbon nanomaterials [6].

Lignin is a highly irregularly branched polyphenolic polyether, consisting of the primary monolignols, p-coumaryl alcohol, coniferyl alcohol and sinapyl alcohol, which are connected via aromatic and aliphatic ether bonds [7] as well as non-aromatic C–C bonds. The high complexity and inhomogeneity of the lignin structure is, in many cases, even further increased by currently applied pretreatment technologies and adds additional challenges for lignin’s downstream processing and valorization [8,9]. Lignins can be roughly distinguished by their origin plant species and the processing method. In case of origin, three major types of lignin can be distinguished; where softwood lignins are comprised almost solely of coniferyl alcohol; hardwood lignins of both coniferyl and sinapyl alcohol; and grass lignins of all three types [10]. In the case of the processing method, two different routes can be identified: (1) lignin recovery before carbohydrate conversion; and (2) lignin recovery after carbohydrate conversion [11]. The latter is expected to be used for low-value markets, whereas lignin removal before fermentation through methods such as organosolv pretreatment or the use of ionic liquids as extraction solvent can result in lignin with little structural changes [11].

A possibility for overcoming the issue of high complexity and inhomogeneity is the use of the lignin at submicron scale, since nanostructured materials can have considerably different properties from larger-dimensional material of the same composition [12]. Production methods for lignin from micro- to nanosize in the shape of particles, fibers, tubes and sheets from different lignin sources were comprehensively compared in a previous review by the authors [13]. However, the lignin materials produced are, in most cases, only intermediates and need to be converted or applied in a final product or process. A summary of applications reported in recent literature is given in the present review.

## 2. Application Overview for Lignin from Micro- to Nanosize

**Figure 1:**
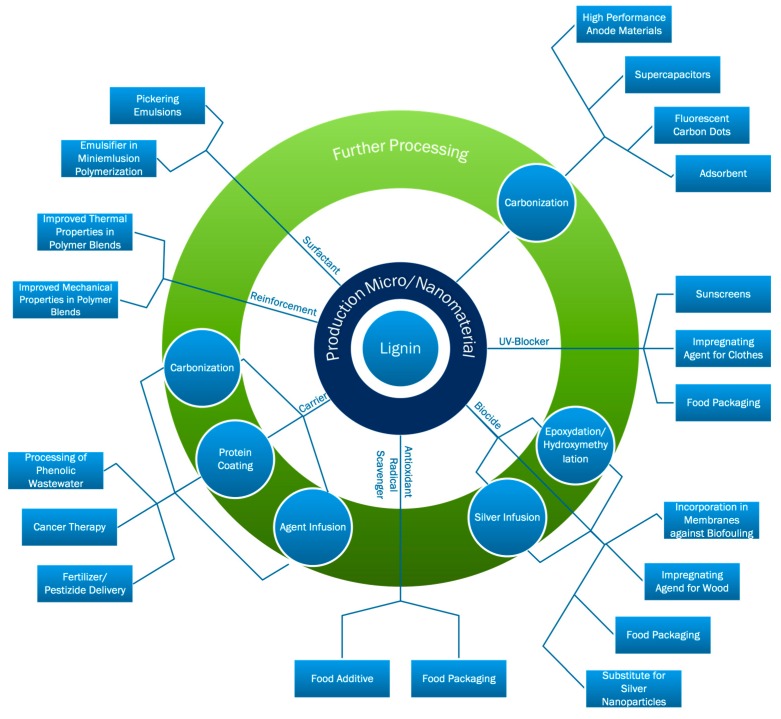
Overview of potential and investigated applications of lignin from micro- to nanosize published in literature.

A wide range of applications for lignin nanomaterials is reported in the literature. This review gives an overview of the present applications and their performance in the final product or process. Figure 1 gives a graphical overview of the published and potential applications.

Lignin shows many unique properties such as resistance to decay and biological attacks, ultraviolet (UV) absorption, high stiffness, and the ability to retard and inhibit oxidation reactions. Therefore, it offers the potential to produce high-value products from a large-volume feedstock [14,15]. Incorporation of nanoparticles in polymers can be a way to increase their values, since current properties can be improved and the materials can be provided with new features. This review is sectioned according to the fields of application of the materials. Therefore, the same polymer blends can be found in several chapters. Table 1 lists all polymer blends in order to give an overview of the fields of applications of polymer blends, the applied production methods, and properties.

## 3. Application of Lignin Nanoparticles (LNPs) as Reinforcement

Nanofillers can improve mechanical properties like stiffness, strength and toughness as well as thermal stability and barrier properties [38]. Those nanocomposites can perform better in comparison to traditional composites due to the higher specific surface of the nanofillers [39]. This section focuses mainly on the mechanical properties, where other properties are discussed in different sections.

Del Saz-Orozco et al. [25] used softwood calcium lignosulfonate particles with an average diameter of 1.6 µm to reinforce phenolic foams. The incorporation of 8.5 wt % of lignin particles in the foam resulted in the greatest mechanical performance and makes it in those terms competitive with synthetic fiber-reinforced phenolic foams. The addition of lignin particles showed a compressive modulus and strength of 128% and 174%, respectively, compared to the neat phenolic foam.

Nevárez et al. [29] incorporated lignin nanoparticles (LNPs) in cellulose triacetate (CAT) membranes and studied the impacts of organosolv lignin (OS), hydrolytic lignin and Kraft lignin (KL) in acetylated and non-acetylated form on the mechanical properties. With only a few exceptions, the mechanical properties were considerably improved through the incorporation of 1 wt % lignin. The hydrolytic lignin, showing the smallest particle sizes among the studied lignins, resulted in the highest Young’s moduli. The membranes can be applied in water purification and might reduce biofouling.

Tensile properties, like the tensile strength (σ), modulus (*E*), and elongation at break (ε*_b_*), are very important indicators for the selection of diverse applications for polymeric formulations [40]. Table 2 shows these parameters for various polymers and their counterpart with incorporated LNPs. The given data shows that the incorporation of LNPs in the right concentration leads to an improved mechanical behavior in all cases.

## 4. Applications of Lignin Nanoparticles as Ultraviolet (UV) Blocker

Lignin contains UV-absorbing functional groups such as phenolic, ketone and other chromophores [42,43]. This UV-absorbing property can be used in applications like suncreens or the impregnation of textiles. The effectiveness of this property can even be improved if the lignin is used at nanoscale. Yearla and Padmasree [44] tested the protection of *Escherichia coli* against UV irradiation by hardwood dioxane lignin and softwood alkali lignin and their complementary nano version, respectively. The UV protectant activity of the nanolignin showed up to a 30% increase in comparison to the non-nanolignin. The UV-absorption spectra of KL nanospheres in an aqueous environment were compared to non-nano KL by Li et al. [45]. The nanospheres with an average hydrodynamic radius of 145 nm showed quite different absorption characteristics compared to the non-nano KL despite their identical chemical composition. Both samples show an absorption maximum at 283 nm, but the nanospherical KL exhibits a broader absorption band in the longer wavelength region and also shows a shoulder towards the longer wavelength region.

Zimniewska et al. [46] treated linen and hemp fabric as well as non-woven flax with nanostructured KL obtained by sonication. The treatment gives the textiles excellent UV-protection while not worsening their physical and bio-physical properties. Using a silicone emulsion for a better fixation of the nanoparticles improved the UV-protection by almost 5 times in comparison with non-treated linen fabric.

Qian et al. [47,48] used lignin from different sources to successfully produce sunscreens. Hydrophobic lignin showed the best performance blended in pure NIVEA^™^ cream, but the synergetic effect with commercial sunscreens must be emphasized. The addition of 10 wt % OS to a commercial sunscreen increased the sun protection factor (SPF) values from 15 to 91.61 and improved its photo stability. In their most recent work, different spherical sizes of the incorporated lignin were investigated, including lignin in nanoscale [49]. It was found that the SPF value of the sunscreens increases with decreasing size of lignin colloidal spheres. While usually, commercial sunscreens need at least 20 wt % chemical sunscreen actives to reach SPF 15, sunscreen containing 10 wt % OS colloidal spheres with the size of about 50 nm reaches the identical SPF value.

**Figure 2:**
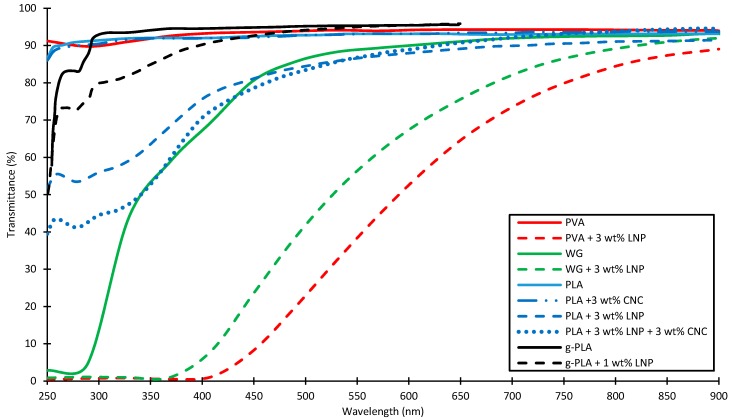
UV–Vis transmission spectra of Polyvinyl alcohol (PVA), Wheat gluten (WG) and Polylactic acid (PLA) polymer blend films with incorporated lignin nanoparticles (LNPs) and cellulose nanocrystals (CNCs). Adapted from [17,19,31,32], with permission from Elsevier.

Yang et al. [17,19,31,32] incorporated LNPs and cellulose nanocrystals (CNC) in wheat gluten (WG), polyvinyl alcohol (PVA), chitosan (CH), glycidyl methacrylate grafted polylactic acid (g-PLA) and neat polylactic acid (PLA). The UV- and visible light-blocking properties of selected blends can be seen on the UV–Vis spectra in Figure 2. Wheat gluten films with incorporated LNPs (0 wt %, 1 wt % and 3 wt %) were prepared with a casting method described by Kayserilioglu et al. [20] and aqueous LNP dispersion was added to the casting solution under vigorous stirring. Incorporation of LNPs decreases the light transmittance in the visible light spectrum indicated by the transmittance at a wavelength of 550 nm, which decreases from 89% for neat WG to 56% for WG containing 3 wt % LNPs. In the UV range (below 400 nm) of the spectra, transmittance below 2% were reached for the LNP containing PVA and WG samples [19]. Binary and ternary polymer films with PVA, CH and LNPs were prepared via solvent casting, and the UV transmittance was reduced from 90.79% to 0.39% for PVA containing 3 wt % LNPs at a wavelength of 320 nm. Ternary mixtures reached transmittance values of 0.87% at 320 nm [32]. PLA films were also produced by extrusion and contained neat PLA, g-PLA and LNPs. The blend consisting of g-PLA and 1 wt % LNPs blocks about 28% of the UV-B (280–315 nm) irradiation [31]. A synergetic effect of incorporated LNPs and CNCs in PLA films was found with respect to their transparency and UV-light blocking capability. Two different amounts of cellulose nanocrystals and lignin nanoparticles were incorporated in a PLA and g-PLA matrix via melt extrusion. The transmittance at a wavelength of 320 nm could be decreased from 91.4% for neat PLA, to 57.8% for PLA with 3 wt % LNP and to 46.2% for PLA including 3 wt % LNP and 3 wt % CNC [17].

## 5. Application of Lignin Nanoparticles as Biocide

Lignin contains different phenolic monomer fragments and is an important source of natural antibacterial compounds. Phenolic fragments containing a double bond in α, β positions of the side chain and a methyl group in the γ position show, in general, the most inhibitory effect [50]. Lignin from different sources, different extraction methods and even a series of lignin model compounds showed successful antimicrobial properties against various microorganisms [51,52,53]. The polyphenolic compounds of lignin cause cell membrane damage and lysis of bacteria with subsequent release of cell content [50,54]. Nanoparticles offer a huge surface area and, therefore, more functional and polyphenolic sidechains on their surface. This increases the contact area and can increase the antimicrobial effect.

Silver nanoparticles, on the other hand, also show effective antimicrobial, antifungal and antiviral properties [55,56]. However, recovery or deactivation of those particles in incineration or wastewater after their intended use is arduous and may adversely affect many ecosystems [57,58,59,60].

Popa et al. [61] and Gîlcă et al. [62] investigated epoxidated and hydroxymethylated lignin nanoparticles as an impregnating agent for wood. Oak, birch and poplar veneer samples were treated with nanoparticle dispersions, buried in garden soil sown with wheat for 6 months and evaluated by their mass loss and variation of contact angle. The birch veneer sample treated with a combination of CuCl and hydroxmethylated sarkanda grass alkali LNPs showed the best results in terms of mass loss. The reference sample showed a mass loss of 80 wt % whereas the treated sample only showed 8.7 wt % [61]. Epoxylated LNPs from commercial Protobind 1000, 2000 and 3000 with average particle diameters of 70–200 nm without addition of CuCl were used for the impregnation of poplar and oak veneer. The mass loss of the untreated reference samples was 37 wt % and 40.1 wt % for poplar and oak, respectively. The best results for oak were achieved by treatment with Protobind 1000 LNPs and a reduction of the mass loss to 20.5 wt %, whereas Protobind 3000 LNPs showed the best result for poplar and reduced mass loss to 9.5 wt % [62]. Those results show improved wood resistance to biodegradation. However, tailored formulations for each wood type seem to be necessary.

Zimniewska et al. [46] impregnated linen fabric with LNPs from Kraft Lignin and achieved antibacterial activity for eight bacteria cultures which are most often found in human environments.

**Figure 3:**
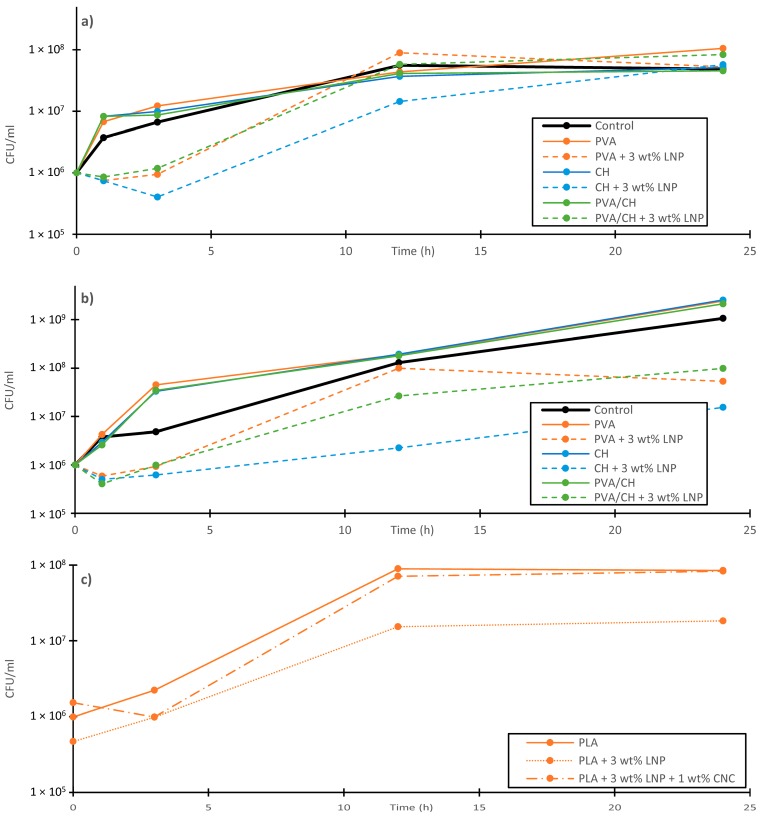
Results of the antimicrobial liquid medium test of polylactic acid (PLA), polyvinyl alcohol (PVA) and chitosan (CH) polymer blends against different bacteria; (**a**) *Pectobacterium carotovorum subsp. odoriferum* (CFBP 1115); (**b**) *Xanthomonas arboricola pv. pruni* (CFBP 3894); (**c**) *Pseudomonas syringae pv. tomato* (CFBP 1323). Adapted from Yang et al. [17,32], with permission from Elsevier.

The antibacterial activity of LNPs incorporated in polymer films for food packaging was evaluated by Yang et al. [17,32]. A liquid medium test [63] was used in all experiments. Two different PLA blends, one containing 3 wt % LNPs and one containing 3 wt % LNPs and 1 wt % CNCs, and neat PLA were tested against the bacterial plant pathogen *Pseudomonas syringae pv. tomato (Pst)*. This bacterium affects tomatoes production worldwide causing serious damage to all organs, including fruits that result unmarketable [64]. In the other study, the biocidal activity of neat PVA, CH, PVA/CH films and nanocomposites containing LNPs was evaluated against *Xanthomonas arboricola pv. pruni* (CFBP 3894) and *Pectobacterium carotovorum subsp. odoriferum* (CFBP 1115) bacterial plant pathogens [32]. Both bacteria can damage fresh fruits and vegetables in open fields and after harvesting [32,65]. The results of investigation can be seen in Figure 3. All LNP-incorporated polymer blends showed lower colony-forming unit (CFU) concentrations within the first 3 h of immersion in the nutrient broth. After 24 h, the *Xanthomonas arboricola pv. Pruni* and *Pseudomonas syringae pv. tomato* concentrations in the tests with incorporated LNPs were significantly lower compared to the neat polymers. However, increasing CFU concentrations were observed in each sample between 3 h and 24 h but remained at lower CFU concentrations compared to the neat polymers [17,32].

**Figure 4:**
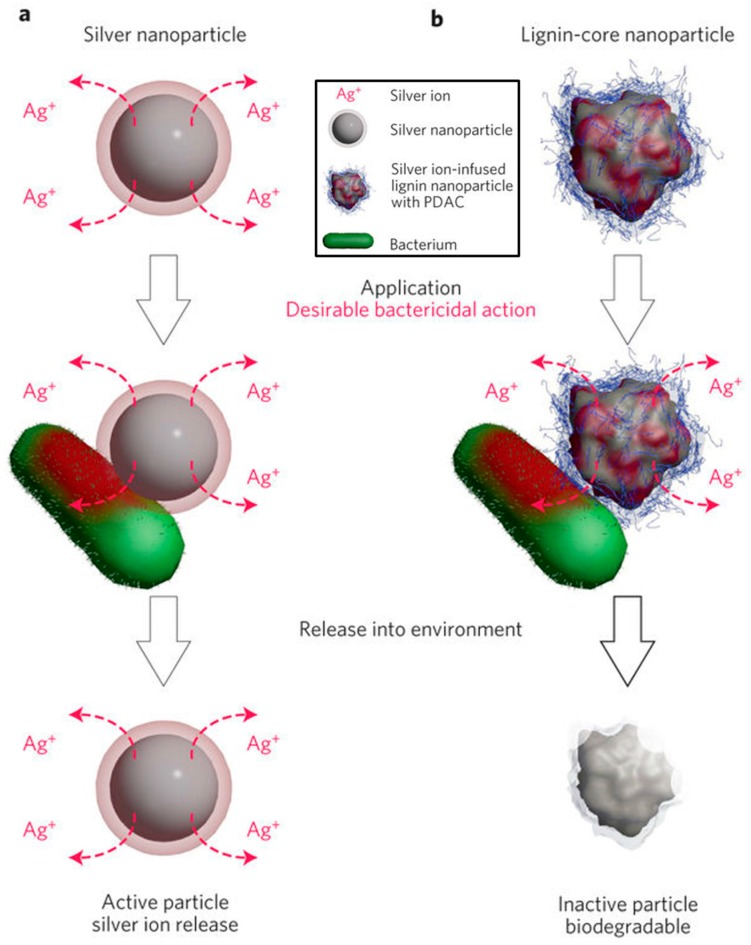
Proposed use cycle and principle of the bactericidal action of environmentally benign lignin-core nanoparticles (**b**) and the currently used silver nanoparticles (**a**). Reprinted by permission from Macmillan Publishers Ltd.: Nature Nanotechnology [66], Copyright 2015.

Richter et al. [66] combined the strong antimicrobial properties of silver with the biodegradability of lignin. Indulin AT™ lignin, obtained by a Kraft pulping process, was used to obtain an environmentally benign core in nanosize. The nanoparticles were precipitated via pH shift and had a mean hydrodynamic diameter of 84 ± 5 nm [67]. Afterwards, particles were infused with Ag^+^ ions in an aqueous solution of AgNO_3_ and coated with polydiallyldimethylammonium chloride (PDAC) to shift the zeta potential of the particles to positive values. Those silver infused particles are capable of neutralizing common Gram-negative and Gram-positive human pathogens as well as quaternary amine-resistant bacteria. The amount of silver used was 10 times less in comparison to conventional silver nanoparticles and aqueous AgNO_3_ solution. Figure 4 shows the proposed schematics of bactericidal activity and behavior of the particles after release into the environment. Both particles release Ag^+^ ions which cause the bacteria to die. The silver-infused lignin particle is supposed to not contain Ag^+^ ions after utilization and remain as a biodegradable lignin particle, whereas the silver nanoparticle still releases Ag^+^ ions after utilization.

## 6. Applications of Lignin Nanoparticles as Antioxidants/Radical Scavengers

Lignin is an effective free-radical scavenger, which can reduce oxygen radicals, and retard and inhibit oxidation reactions [68,69]. Most antioxidant effects of lignins are considered as derived from the scavenging action of their phenolic structures on oxygen-containing reactive free radicals. Therefore, the reducing power of a compound may serve as a significant indicator of the potential antioxidant activity [70]. The scavenging activity is predominantly based on non-etherified phenolic hydroxyl groups, ortho-methoxy groups and aliphatic hydroxyl groups in the side chain [69].

**Figure 5:**
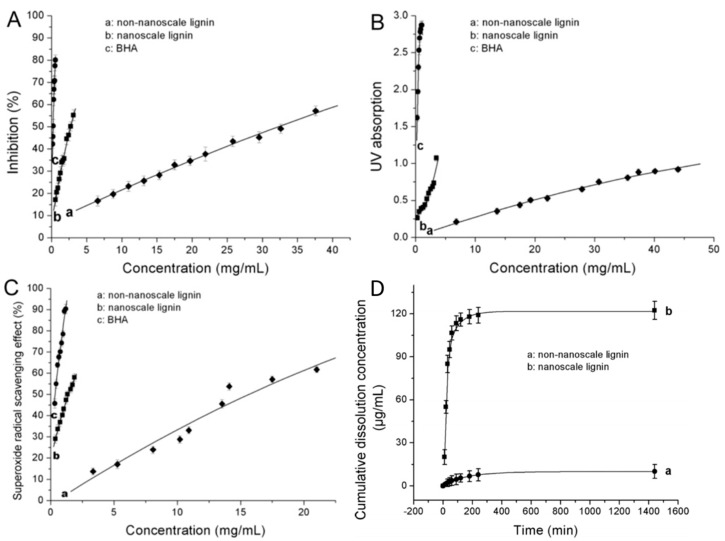
(**A**) 2,2-diphenyl-1-picrylhydrazyl (DPPH) radical-scavenging capacity; (**B**) reducing power; (**C**) superoxide radical scavenging activity; and (**D**) Dissolution profiles in water at room temperature of non-nanoscale lignin and nanoscale lignin. The antioxidant butylated hydroxyanisole (BHA) was used as control. Reprinted from [71], with permission from Elsevier.

Lu et al. [71] compared the 2,2-diphenyl-1-picrylhydrazyl (DPPH) radical-scavenging activity (RSA), reducing power, superoxide radical-scavenging activity (SRSA) and water solubility of nanoscale poplar OS (144 ± 30 nm) in comparison with non-nanoscale lignin. The chemical of structure of nanoscale lignin did not change compared to the non-nanoscale lignin. The results in Figure 5 show improved RSA, reducing power and SRSA for the LNPs in comparison to the non-nanoscale lignin. Surprisingly, the solubility increased more than 12-fold despite the same chemical composition. Further improvement of the RSA is possible by adaptation of the lignin extraction parameters, as indicated by Pan et al. [72] where ethanol OS from hybrid poplar with more phenolic hydroxyl groups, less aliphatic hydroxyl groups, low molecular weight, and narrow polydispersity showed high antioxidant activity. These properties were improved at elevated temperature, longer reaction time, increased catalyst concentration, and low ethanol/water ratio in the pretreatment process.

The improvement of RSA depending on the particle size of hardwood dioxane lignin and softwood alkali lignin was investigated by Yearla et al. [44]. The spherical LNPs had mean diameters ranging from 80 nm to 104 nm. The RSA of the LNPs, however, showed only marginally differences when compared to their non-nanoscale lignins where the alkali lignin showed better performance compared to the dioxane lignin. These results are contrary to the findings of Lu et al. [71].

Ge et al. [73] investigated the free RSA of alkaline lignin LNPs produced by two different methods. The first using ethylene glycol as a solvent, and the second using alkaline water as shown in previous work for sugarcane bagasse [74]. The IC_50_ (dosage of lignin at free RSA = 50%) of each lignin was calculated, where a higher antioxidant activity is indicated by a lower IC_50_ value. The IC_50_ values are 0.18 ± 0.01 mg/mL, 0.30 ± 0.02 mg/mL and 0.60 ± 0.05 mg/mL for LNPs precipitated by the second method, non-nanoscale lignin and LNPs precipitated by the first method, respectively. This indicates a dependency of the free RSA on the precipitation method of the LNPs.

Yang et al. [32] investigated the DPPH RSA for binary and ternary PVA/CH nanocomposites containing LNPs for active food packaging following the methods reported by Byun et al. [75] and Domenek et al. [76]. Neat PVA was used as control material. Nanocomposites containing 1 wt % LNPs reached a RSA of up to 78.2% and samples containing 3 wt % up to 92%.

## 7. Application of Lignin Nanoparticles as Surfactants in Pickering Emulsions

Surfactants consist of polar head groups and nonpolar tails and are categorized by the chemistry of their headgroup, including anionic species based on fatty acids and sulfonate derivatives [77], cationic species based on arginine lipopeptides [78], and nonionic species based on alkyl polyglycosides [79]. Unlike traditional surfactant-stabilized emulsions, Pickering emulsions are stabilized by solid particles at the interface between the dispersed phase and the continuous phase [80]. The generally large energetic barrier associated with the particle desorption from the interface makes them inherently more stable against coalescence than their surfactant-based counterparts [81].

Nypelö et al. [82] stabilized hexadecane in water emulsions with KL particles with a size of about 320 nm. The hexadecane droplets in the Pickering emulsion had average diameters of 7.7 ± 1.6 µm and 22.3 ± 3.6 µm for 0.6 wt % and 0.2 wt % particle concentrations, respectively. The emulsions were stable over the observation period of one week.

**Figure 6:**
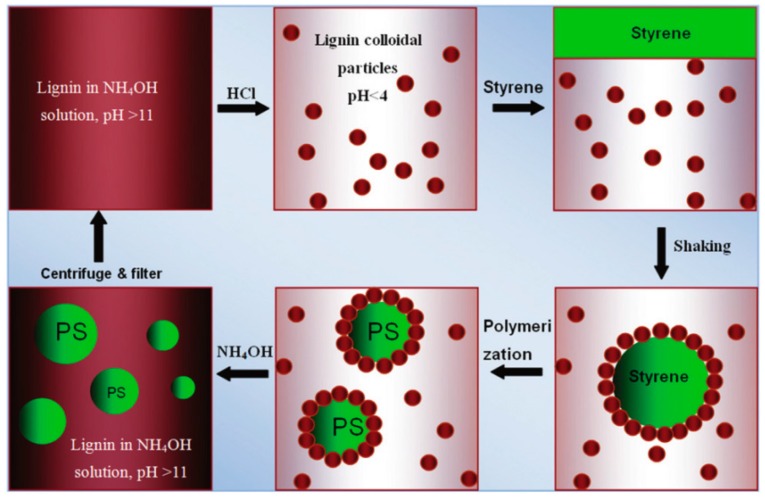
A schematic representation of the preparation of lignin-coated microparticles and bare polystyrene (PS) microparticles based on a lignin-stabilized Pickering emulsion including recirculation of lignin. Reproduced from [83] with permission of The Royal Society of Chemistry.

Wei et al. [83] used alkaline lignin as auxiliary material in the production of polystyrene (PS) microparticles (see Figure 6). Under acidic conditions at pH 4, lignin forms particles with an average size of 182 nm. These particles form a Pickering emulsion with the styrene/water. Styrene droplets are surrounded by the lignin particles and droplet size can be adjusted by the concentration of lignin particles. Droplet sizes from 22.31 μm to 58.93 μm were achieved for lignin concentrations based on water from 1 wt % to 0.05 wt %. After polymerization of the styrene, lignin was recovered by raising the pH value and dissolving the lignin particles.

Nanoparticles grafted with surface active polymer chains can decrease surface and interfacial tensions and create stable Pickering emulsions [84,85,86]. Grafting properties like graft density, length and architecture can be controlled by techniques such as atom transfer radical polymerization (ATRP) [87], nitroxide-mediated radical polymerization (NMP) [88], and reversible addition–fragmentation chain transfer (RAFT) [89,90].

Table 3 shows the grafting methods and polymers used for the applications of grafted LNPs in Pickering emulsions. Gupta et al. [91] could form stable water-in-oil emulsions where the poly(acrylic acid) grafted LNPs performed significantly greater than polyacrylamide grafted ones regarding the partitioning into the emulsion phases. Emulsions were stable for weeks at room temperature. The solution viscosities did not increase significantly by adding grafted LNPs while the surface tensions decreased as a function of concentration, graft density, graft molecular weight and polymer chemistry. Silmore et al. [80] could produce Pickering emulsions stable for a period of months with only 0.1 wt % nanoparticle content. The interfacial tension was found to decrease with decreasing graft density and increasing salinity.

**Figure 7:**
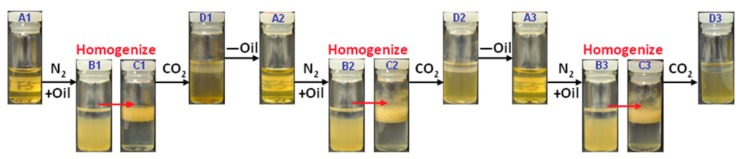
Illustration of three cycles of CO_2_-triggered emulsification and N_2_-triggered demulsification of lignin-g-DEAEMA water/decane Pickering emulsion. Reproduced from [92] with permission of The Royal Society of Chemistry.

Qian et al. [92] developed a Pickering emulsion offering a gas-switchable feature. Figure 7 shows the highly reversible and repeatable process of dispersing decane in water by CO_2_ bubbling and precipitation by N_2_ bubbling. The dispersion and precipitation ability is determined by the grafting density and chain length of the 2-(diethyl-amino)ethyl methacrylate (DEAEMA) units where shorter chain length resulted in faster flocculation of the grafted lignin when switching to N_2_ bubbling. The diameters of the produced particles showed a range of 237–404 nm, and concentration of 1 g/L grafted lignin based on the water content was sufficient to gain Pickering emulsions with a water-to-decane volumetric ratio of 4:1.

## 8. Applications of Carbonized Lignin Nano Fibers and Particles

Carbon nanostructures such as fibers and particles offer numerous possibilities for applications like batteries, supercapacitors, fuel cells, structural composites, filtration devices. Fluorescence carbon nanoparticles can be applied for biological labeling, bioimaging, and other optoelectronic device applications. Therefore, this topic is rapidly gaining interest in research [93,94,95,96]. Lignin can be used as a precursor for those carbon fibers and particles and opens application possibilities in high-value products. Carbon fibers from commercially available KL were already successfully produced by Kadla et al. [97] in 2002. Increasing tensile strength and modulus was observed with decreasing fiber diameter where the smallest diameters of about 30 µm were achieved. This leads to the assumption that further reduction of diameters to nanoscale could significantly improve these properties.

Hu and Hsieh [98] produced highly porous activated carbon fibers by the electrospinning of aqueous solutions of predominantly spruce wood alkali lignin followed by simultaneous carbonization and activation at 850 °C under an N_2_ atmosphere. These carbon fibers with diameters from 100 to 500 nm were fabricated into supercapacitors for capacitive energy storage and demonstrated outstanding electrochemical performance [99]. The capacitators reached an energy density of 8.1 Wh/kg based on the averaged electrode mass and over 96% capacitance-retention rates were achieved after 5000 charge/discharge cycles. Also, Lai et al. [100] used carbon nanofiber mats produced from electro-spun alkali lignin in high-performance supercapacitors. In this study, mixtures of alkali lignin/polyvinyl alcohol (PVA) were used and resulted after electrospinning in average fiber diameters of about 100 nm. The capacitance retention was retained to approximately 90% after 6000 cycles of charge/discharge.

Wang et al. [101] utilized OS/polyethylene mixtures for the production of carbon fibrous mats dedicated for use as high-performance anode material in lithium-ion batteries. The production process involved electro spinning followed by carbonization and thermal annealing in the presence of urea. The fibrous mats achieved specific capacities up to 445 mAh/g, which are comparable to currently used polyacrylonitrile-derived carbon fibers.

Gonugunta et al. [102] successfully synthesized carbon nanoparticles from commercial Protobind 2400 Lignin by freeze drying the lignin solution and through carbonization of the resulting lignin nanoparticles in a tube furnace. The process yielded porous particles in the size range of 25 to 150 nm.

Yiamsawas et al. [103] methacrylated KL, which was subsequently processed to particles with an extraordinary porous structure. The particles with diameters up to 2–3 μm were prepared in the absence of surfactants or in the presence of the surfactant Lutensol AT25 and were carbonized at 600 °C and 800 °C. Structural parameters were analyzed by the BET method (see Table 4). The adsorption ability, which is the property mainly used in applications, was evaluated by using methylene blue as a model adsorbent. Carbon particles prepared from porous LNPs showed an adsorption capacity twice as high as the carbon particles prepared from pristine lignin.

**Figure 8:**
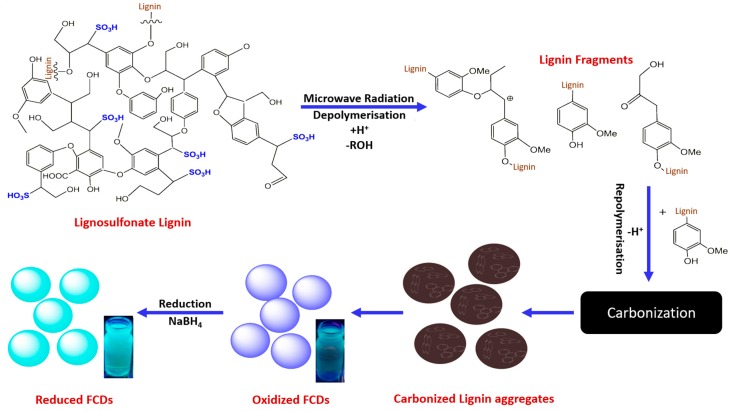
Schematic representation of the production procedure for reduced fluorescence carbon dots from lignosulfonate lignin in aqueous acidic media, including possible depolymerisation and formation reaction mechanisms in which sulfonate groups were neglected. Reprinted from [107], with permission from Elsevier.

Carbon dots are, due to their optical stability, low toxicity, biocompatibility and ease of functionalization, an interesting class of carbon nanoparticles [104,105,106]. Rai et al. [107] synthesized reduced fluorescence carbon dots (rFCDs) from lignosulfonate lignin using a microwave irradiation method previously described by Chen et al. [108]. This method includes a depolymerisation step in order to reach particle sizes of less than 10 nm and differs, therefore, from other production methods. A brief schematic representation of the whole process is given in Figure 8. The process resulted in cost-effective water-soluble rFCDs for bioimaging applications. The photoluminescent intensity maximum was found at 475 nm when excited with 440 nm.

## 9. Application of Lignin Nanoparticles as Nano- and Microcarrier

Currently, based on the number of published research papers, the use of lignin nanostructures in the medical field as drug carriers seems to be the most investigated field of application. Generally, the nanocarriers can be distinguished in two categories: (1) hollow particles where the carried substance is encapsulated; (2) solid and porous particles infused with the carried substance.

### 9.1. Capsules, Hollow and Porous Structures

In recent years, microencapsulation processes have gained significant interest in academic and industrial research [109,110,111,112,113,114,115]. Microencapsulation has potential applications in the delivery of soluble drugs [116,117], pesticides [118] and gene [119,120] and food additives [121]. The major problem for controlled release systems is the initial burst which releases a great amount of active agent causing an acutely high concentration and therefore, a failure of the controlled release [122,123]. Encapsulation might be key for this issue.

**Figure 9:**
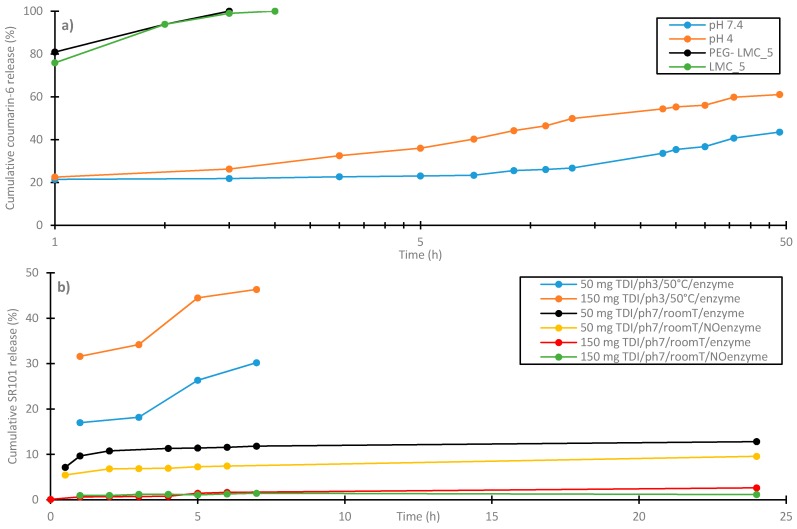
Releasing rates of hydrophobic Coumarin-6 and hydrophilic SR101 for different lignin capsules. (**a**) Coumanrin-6 releasing rate of capsules produced by Chen et al. [126] at pH 4 and pH 7.4 (Adapted with permission from [126]. Copyright 2016 American Chemical Society) and capsules (PEG-LMC_5 and LMC_5) produced by Tortora et al. [125] (Adapted with permission from [125]. Copyright 2014 American Chemical Society); (**b**) SR101 releasing rate at different amounts of crosslinking agent TDI, pH values, temperatures and degradation by enzymes [124], published by The Royal Society of Chemistry.

Lignin nano- and microcapsules for the transport of hydrophobic and hydrophilic substances with different releasing properties via miniemulsion polymerization are reported in literature [103,124,125,126]. Chen et al. [126] used allyl-grafted lignosulfonate to prepare capsules via ultrasonication in an oil-in-water miniemulsion. The method resulted in capsules with diameters ranging from 50 to 300 nm. For the release study, the hydrophobic model drug coumarin-6 was incorporated in the oil particle core by dissolving it in the oil phase before sonication. The capsules showed an entrapment capacity of 0.713 mmol/g based on the weight of modified lignosulfonate. The release study was conducted in aqueous surfactant-containing buffer solutions at pH 4 and pH 7.4. The release kinetics showed an enhanced release rate at the lower pH value (see Figure 9a; dataset pH 7.4 and pH 4). This behavior might be ascribed to the destruction of acid-liable β-thiopropionate cross-linkages [127,128] and weakening of the hydrophobicity of lignin’s cross-linked shell by protonation of coumarin-6 at low pH-values [129]. Also, Tortora et al. [125] conducted a release study of coumarin-6 on lignin capsules. In this study, pure KL was used to produce capsules in a size range of 0.3 to 1.1 µm depending on the cross-linking agent used. The addition of poly(ethylene glycol) diglycidyl ether (PEG) and H_2_O_2_ reduced the size of the capsules in comparison with the use of just sonication, where PEG achieved the smallest sizes. The biocompatibility study of theses capsules with Chinese hamster ovary cells shows promising results for a potential use in the biomedical field. The release study was conducted in water using the same surfactant and surfactant concentration as used by Chen et al. [126] but the pH value was not controlled by a buffer. Figure 9a shows also the release kinetics of the capsules made without cross-linking agent (LMC_5) and made with PEG (PEG-LMC_5). Both kinds of capsules released their cargo at a similar rate where almost 100% of entrapped Coumarin-6 was released after 1 h. However, the release rate differed significantly from the rates obtained by Chen et al. [126] which showed a maximum release of 60% after 48 h.

Yiamsawas et al. [124] used interfacial polyaddition in inverse miniemulsions to prepare nanocapsules for hydrophilic loadings. Lignosulfonic acid sodium salt with 2,4-Toluene diisocyanate (TDI) as cross-linker resulted in capsules in a range of 150–200 nm. A releasing study with hydrophilic fluorescent dye sulforhodamine (SR101) including the investigation of the biodegradability was conducted. The cleavage of the cross-linked lignin shell was studied by using a laccase enzyme from the fungus *Xylaria* sp. IBWF A55-2009, which has an important role in the recycling of lignin [130]. Figure 9b shows the releasing kinetics of SR101-filled capsules with different amounts of cross-linking agent TDI at pH values of 3 and 7 and with and without degradation by enzymes. The results clearly show that less crosslinking agent, higher temperatures, lower pH values and degradation by enzymes increase the release rate.

**Figure 10:**
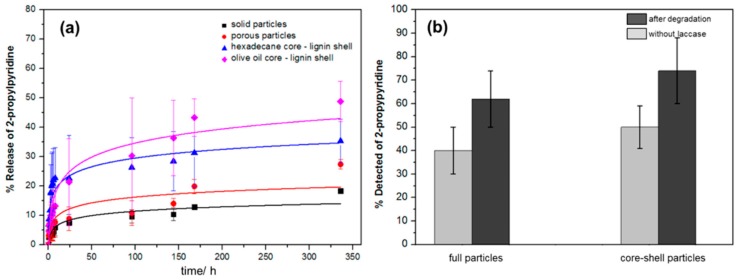
(**a**) Release profile of 2-propylpyridine from lignin nanocarriers with varying morphology, core or surfactant. (**b**) Amount of 2-propylpyridine released after 24 h with and without enzymatic degradation of solid and core–shell nanocarriers with enzymes after 24 h. Reprinted with permission from [103]. Copyright 2017 American Chemical Society.

In a further work, Yiamsawas et al [103] was producing nano- and microparticles with variable morphologies using KL modified by esterification with methacrylic anhydride in a combined miniemulsion polymerization, with subsequent solvent evaporation. The solid nanoparticles, nanocapsules and porous microparticles were loaded with 2-propylpyridine and release was studied following the method of Dowding et al. [131]. 2-propylpyridine was used since it is soluble in the oil phase and partially soluble in the aqueous phase. Figure 10a shows the release study results. Solid and porous particles show the lowest rates whereas particles with an oil core and lignin shell show a higher rate. In addition, the type of oil used as core seems to influence the releasing rate. Studies with degradation, using the enzyme laccase, were conducted and indicate a higher releasing rate when treated with enzymes (see Figure 10b).

Ten et al. [132] synthesized lignin nanotubes (LNT) from different sources using a sacrificial alumina membrane template. Those nanotubes are intended to be used in the medical therapeutic field and to substitute carbon nanotubes (CNT) whose use has caused concerns due to their cytotoxicity [133,134]. Cytotoxicity studies with human cervical adenocarcinoma (HeLa) cells showed a toleration threshold 10 times higher when compared to CNTs. DNA can successfully adsorb on the LNTs and leads to transfection in HeLa cells. Furthermore, it appears that the lignin-isolation procedure is mainly influencing the cytotoxicity, and that the transfection efficiency is also influenced by the lignin source.

Zhong et al. [135] encapsulated the enzyme horseradish peroxidase in sodium lignosulfonate reverse micelles. Free enzymes easily lose their activity when exposed to the natural environment whereas immobilized enzymes can gain stability, adjust reactions, and improve recyclability. The immobilized enzyme showed enhanced activity compared to free horseradish peroxidase at acidic and low-temperature environments. Those properties might allow applications in the processing of phenolic wastewater due to the catalytic polymerization property of horseradish peroxidase being now viable under harsher conditions.

A layer-by-layer method was used by Wand and Zhao [136] in order to encapsulate the herbicide picloram (PLR) with alternating layers of chitosan and sodium lignosulfonate. The release time of the PLR and its photo stability was adjustable according to the number of layers used. The time required for the release of 50% PLR from the capsules was 0.433 h, 1.015 h and 2.257 h for 4, 8 and 12 layers, respectively.

### 9.2. Solid and Porous Particles/Structures

In the case of solid particles, the active agent is incorporated in the LNPs already during the precipitation step as shown by Figueiredo et al. [137] and Dai et al. [138].

**Figure 11:**
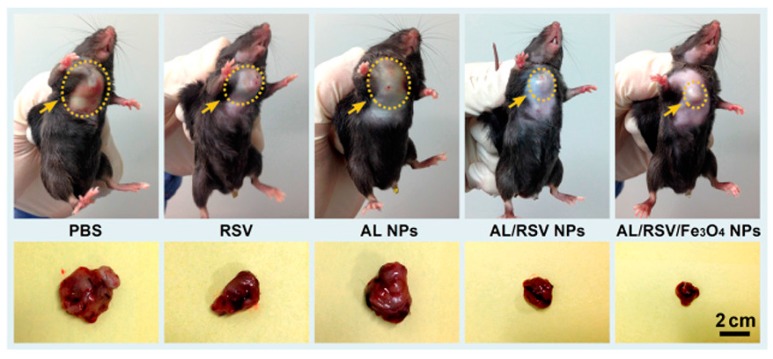
Tumor photographs of mice treated with different nanoparticles, pure Resveratrol and phosphate-buffered saline (PBS) as control. AL/RSV/Fe_3_O_4_, an LNP containing Resveratrol and Fe_3_O_4_ showed the best performance and could reduce the tumor size significantly. Adapted with permission from [138]. Copyright 2017 American Chemical Society.

Dai et al. [138] precipitated LNPs using ethanol, methanol and tetrahydrofuran (THF) as solvent and water or aqueous Fe_3_O_4_ nanodispersion as antisolvent. Resveratrol (RSV), a bioactive molecule, was used as active ingredient. In order to incorporate RSV in the LNP, it was mixed with the solvent and incorporated during the precipitation process and resulted in an average hydrodynamic LNP diameter of 180 nm. Cytological and animal tests were conducted and showed good anticancer effects, enhanced by in vitro RSV release, stability and accumulation and better tumor reduction (see Figure 11). Also, the LNPs had a relatively high drug-loading capacity of over 20 wt % as well as reduced adverse effects compared to free RSV.

Figueiredo et al. [137] used similar methods for the preparation of Fe_3_O_4_ LNPs, but the precipitation step was conducted via a dialysis process. The cytotoxic test agents, sorafenib (SFN) and benzazulene (BZL), and a water-soluble drug, capecitabine (CAP), were also incorporated in the LNPs during the precipitation process. All LNPs exhibited low cytotoxicity at concentrations up to 100 mg/mL in different cell lines. Overall, these LNPs showed important features for drug delivery and biomedical applications, including biocompatibility, very good stability, an ability to load hydrophobic drugs and sustain their release, and good cellular interaction. Furthermore, the particles including Fe_3_O_4_ showed superparamagnetic behavior, which makes them promising for magnetic targeting and magnetic-resonance imaging.

## 10. Conclusions and Outlook

Different application fields of lignin from micro- to nanosize were identified from the literature ranging from simple polymer blends with improved mechanical properties to promising drug carriers for cancer treatment. Consequently, the possibility of applying lignin in high-value pharma fields was also shown. The parameters influencing the properties of the final product include the lignin source, the method of extracting lignin from the lignocellulose raw material, the production/precipitation method of the nanoparticles which influences the surface properties of the particles and, in the case of polymer blends, the processing/blending method.

This is clearly an opportunity to tailor the properties of the final product by optimizing the whole process chain. On the other hand, considering a whole biorefinery in which switching raw materials and lignin-extraction methods is not easily possible, it is crucial to determine suitable final products for existing processes. Moreover, changing raw lignocellulose quality might influence the LNP and final product quality. Nevertheless, a large number of potential high-value applications are within the range of vision for micro- and nanosize lignin particles.

## Figures and Tables

**Table 1:** Overview of investigated polymer blends.

Investigated in	Neat Polymer	Production Method	Application Field and Neat Properties	Investigated Properties by Addition of Lignin Nanoparticles (LNPs)
[16,17]	Polylactic acid (PLA)	- Extrusion - Solvent casting	- Widely used as a packaging material - Poor thermal and mechanical properties; poor ultraviolet (UV) barrier properties [18]	- UV-transmittance - Antibacterial activity - Mechanical properties - Thermal properties
[19]	Wheat gluten (WG)	- Solvent casting as described by Kayserilioglu et al. [20] - Addition of aqueous LNP suspension to the casting solution	- Can be easily processed into films; effective barrier properties against carbon dioxide, oxygen and aroma components, rapidly biodegradable [21,22,23,24] - Poor mechanical properties; strong water absorption in humid environment	- UV-transmittance - Mechanical properties - Thermal properties - Water sensitivity
[25]	Phenolic foams	- Mixing dry lignin particles with resol resin - Mixing of resol resin, surfactant, catalyst, hardener and blowing agent	- Low thermal conductivity; exceptional fire-resistant properties; high thermal stability; low cost [26] - Relatively low mechanical performance; extreme friability compared to other polymeric foams [27,28]	- Mechanical properties
[29]	Cellulose triacetate (CTA)	- Solvent casting - Separate solving of CTA and LNPs in methylenchloride	- Membrane production - Low mechanical strength; poor resistance to oxidation [30]	- Mechanical properties
[31]	Glycidyl methacrylate grafted PLA	- Extrusion	- Widely used as a packaging material - Poor thermal and mechanical properties; poor UV barrier properties [18]	- UV-transmittance - UV-weathering stability - Mechanical properties - Thermal properties
[32,33]	Poly vinyl alcohol (PVA)/chitosan (CH)	- Solvent casting	- Widely used in food packaging, biomedical, household and construction sector - Good solvent resistance, mechanical performance und biocompatibility [34,35] - High moisture absorption; low biodegradation rate	- UV-transmittance - Thermal properties - Mechanical properties - Migration - Antioxidant activity - Antibacterial activity
[36]	Bio-poly (trimethylene terephthalate)	- Extrusion	- Good tensile behavior, outstanding elastic recovery and thermal stability [37]	- Thermal properties - Mechanical properties - Biodegradation

**Table 2:** Mechanical properties of different neat polymers in comparison to their nanocomposites.

Source	Polymer	Filler Composition and Concentration	Tensile Strength σ (MPa)	Young’s Modulus *E* (MPa)	Elongation at Break *ε_b_* (%)
[19]	Wheat gluten	neat	5.5 ± 0.8	180.5 ± 57.9	297 ± 11
		3 wt % LNP	13.3 ± 1.6	553.2 ± 56.3	28 ± 10
[32]	PVA	neat	45.7 ± 1.3	1140 ± 220	164 ± 15
		3 wt % LNP	51.4 ± 3.3	2100 ± 130	30.6 ± 8.5
[17]	PLA	neat	44.0 ± 4.6	2010 ± 210	15.0
		3 wt % LNP	41.1 ± 1.9	1390 ± 60	66.3
		1 wt % LNP + 3 wt % CNC	53.6 ± 6.9	2500 ± 170	7.3
[31]	PLA/g-PLA	neat PLA	44.4 ± 4.3	1950 ± 250	16.9 ± 4.0
		PLA + 1 wt % LNP	48.6 ± 3.5	2150 ± 130	26.8 ± 4.8
		g-PLA+ 1 wt % LNP	47.2 ± 3.1	1630 ± 110	108 ± 20
		PLA/g-PLA + 1 wt % LNP	56.4 ± 3.3	2120 ± 140	20.4 ± 8.9
[16]	PLA	neat	44.3 ± 4.6	1960 ± 230	17.0 ± 3.8
		PLA + 1 wt % LNP	48.6 ± 3.4	2150 ± 130	26.7 ± 4.8
		PLA + 3 wt % LNP	40.9 ± 2.1	1380 ± 60	66.7 ± 4.0
[41]	Natural rubber	neat	25.24 ± 0.38	2.00 ± 0.03 ^2^	654 ± 13
		7 wt % LNP ^1^	29.24 ± 0.59	2.95 ± 0.10 ^2^	658 ± 20
[36]	bio-PTT	neat	51.49 ± 0.5	2058 ± 37	-
		1.5 wt % LNP	59.16 ± 0.7	2227 ± 47	-
		1.5 wt % LNP + 7 wt % carbon fibers	61.74 ± 0.6	2309 ± 9	-

^1^ Based on the weight of lignin in the particles; ^2^ at 300% elongation not at break.

**Table 3:** Overview of the grafted LNPs for use in Pickering emulsions.

Source	Lignin Type	Grafting Method	Grafted Polymers	Stabilized Emulsion
[91]	KL	RAFT	polyacrylamide	water/hexanes ^1^
			poly(acrylic acid)	
[80]	KL	RAFT	polyacrylamide	water/cyclohexane
[92]	AL	ATRP	2-(diethyl-amino)ethyl methacrylate	water/decane

^1^ mixture of C_6_H_14_ isomers as well as methylcyclopentane.

**Table 4:** Oxygen and carbon composition determined by X-ray photoelectron spectroscopy (XPS) and structure parameters obtained by the Brunauer–Emmett–Teller (BET) method for samples before and after carbonization at different temperatures. Adapted with permission from [103]. Copyright 2017 American Chemical Society.

Sample	Carbonization Temperature (°C)	O (%)	C (%)	BET Surface Area (m^2^/g)	Average Pore Volume (cm^3^/g)	Average Pore Width (Å)
Pristine lignin	-	27	73	5	0.017	16.5
Porous LNP	-	21.6	78.4	15	0.020	34.7
Pristine lignin	800	14.1	85.9	29	0.023	18.5
Porous LNP	600	17.2	82.8	202	0.123	14.7
Porous LNP	800	8	92	552	0.274	14.8

## Abbreviations

AL	Alkali lignin
ATRP	Atom transfer radical polymerization
BHA	Butylated hydroxyanisole
Bio-PTT	Bio-poly(trimethylene terephthalate)
BZL	Benzazulene
CAP	Capecitabine
CAT	Cellulose triacetate
CFU	Colony-forming unit
CH	Chitosan
CNC	Cellulose nanocrystals
CNT	Carbon nanotube
DEAEMA	2-(diethyl-amino)ethyl methacrylate
DPPH	2,2-diphenyl-1-picrylhydrazyl
g-PLA	Glycidyl methacrylate grafted polylactic acid
HeLa	Human cervical adenocarcinoma
KL	Kraft lignin
LNP	Lignin nanoparticles
LNT	Lignin nanotubes
NMP	Nitroxide-mediated radical polymerization
OS	Organosolv lignin
PBS	Phosphate-buffered saline
PDAC	Polydiallyldimethylammonium chloride
PEG	Poly(ethylene glycol) diglycidyl ether
PLA	Polylactic acid
PLR	Picloram
PS	Polystyrene
PVA	Polyvinyl alcohol
RAFT	Reversible addition–fragmentation chain transfer
rFCDs	Reduced fluorescence carbon dots
RSA	Radical scavenging activity
RSV	Resveratrol
SFN	Sorafenib
SPF	Sun protection factor
SR101	Hydrophilic fluorescent dye sulforhodamine
SRSA	Superoxide radical scavenging activity
TDI	2,4-Toluene diisocyanate
WG	Wheat gluten
